# Technology Acceptance Model in Medical Education: Systematic Review

**DOI:** 10.2196/67873

**Published:** 2025-07-16

**Authors:** Jason Wen Yau Lee, Jenelle Yingni Tan, Fernando Bello

**Affiliations:** 1 Duke-NUS Medical School, National University of Singapore, 8 College Road, Singapore, 169857, Singapore, 65 66016437; 2 Faculty of Arts and Social Science, National University of Singapore, Singapore, Singapore; 3 Imperial College, London, United Kingdom

**Keywords:** technology acceptance model, medical education, systematic review, TAM, learners, educators, technologies, technology, theoretical framework, technology adoption, electronic literature, qualitative, survey instrument, surveys, technology acceptance, learning interventions, e-learning, mobile learning

## Abstract

**Background:**

With the growing use of technology in medical education, a framework is needed to evaluate learners’ and educators’ acceptance of these technologies. In this context, the Technology Acceptance Model (TAM) offers a valuable theoretical framework, providing insights into the determinants influencing users’ acceptance and adoption of technology.

**Objective:**

This review aims to systematically synthesize the body of research in medical education that uses the TAM.

**Methods:**

An electronic literature search was conducted using the PRISMA (Preferred Reporting Items for Systematic Reviews and Meta-Analyses) approach in February 2024 on the Embase, MEDLINE, PsycINFO, PubMed, and Web of Science databases, yielding 680 articles. Upon elimination of duplicates and applying the exclusion criteria, a total of 39 articles were retained. To evaluate the quality of the study, the Medical Education Research Study Quality Instrument score was calculated for each analysis with a qualitative component.

**Results:**

Studies using TAM in medical education began in 2010, with the model’s application relatively rare up to 2016. Most of the studies were quantitative, operationalizing the TAM as a survey instrument, but it was also used as a research framework in qualitative data analysis. Structural equation modeling, descriptive analysis, and correlation analysis were the most common data analysis approaches in the studies. E-learning and mobile learning were the predominant learning interventions explored, but there were indications that novel learning technologies such as augmented reality, virtual reality, and 3D printing were being investigated.

**Conclusions:**

The study’s findings reveal an expanding scholarly engagement with using TAM in medical education. Although the TAM has been mostly used as a survey instrument, it can also be adapted as a qualitative research framework to analyze data. This systematic review provides a foundation for future research to understand the factors influencing users’ acceptance of technology, especially in medical education.

## Introduction

Technology has changed how we learn and access knowledge, particularly with the introduction of digital devices and the internet. No longer are we constrained by time or space, and information is available anytime and anywhere. Today, learning can happen through massive open online courses such as Khan Academy [1], edX [2], and Coursera [3], or simply by viewing one of countless video tutorials online. Books can be supplemented or even replaced with multimedia resources that can provide learners with a richer learning experience. The way that knowledge is accessed has changed dramatically over the past 2 decades with the development of new technologies.

Medical education has traditionally relied on time-honored teaching methodologies. Cadaveric dissection has always been considered the gold standard for anatomy instruction [4], providing students with hands-on experience with human tissues and structures. However, as educational resources face constraints and medical knowledge expands, these traditional approaches have begun to be transformed through the use of technology. Teaching modalities such as virtual [5] and augmented reality [6] provide students with an immersive 3D learning experience. Three-dimensional printing technology [7] has enabled the creation of anatomical models on demand that can be customized for specific learning outcomes [8], and e-learning resources [9] have democratized access to high-quality learning materials. In a study on rural posting clerkships, iPads equipped with mobile health information resources have positively influenced medical students’ information-seeking behavior [10]. With the increasing use of technology in medical education, it is essential to understand how it is accepted for use in learning.

The Technology Acceptance Model (TAM) provides a framework to understand the factors influencing the decision to use new technologies in medical education [10-12]. The perceived ease of use is the extent to which a person believes the system will be free of effort. In contrast, perceived usefulness is the extent to which a person believes using the system would improve their productivity or job performance. However, one shortcoming of the TAM when applied to complex medical teaching environments is that it does not consider broader contextual factors, such as organizational culture, social influence, and other affective factors like attitudes and beliefs that may significantly impact the acceptance of educational technology.

To address this issue, the Technology Acceptance Model 2 (TAM2) was proposed by Venkatesh and Davis [13] as an improvement to the original model to include social influence and cognitive processes that may influence an individual’s acceptance of technology. The purpose of developing the TAM2 was to include additional crucial factors influencing perceived usefulness and usage intention constructs to explain user behavior and acceptance. These factors include subjective norms, output quality, result demonstrability, and social factors, among others, that explain user behavior and acceptance. By integrating these factors, the TAM2 offers a more comprehensive framework for analyzing how individual and social variables influence beliefs, attitudes, and intentions to use the technology in medical education.

Despite the growing use of technology in medical education, understanding the factors that influence its adoption remains challenging for educators and institutions. Previous research has identified barriers to technology implementation such as technical difficulties [14], resistance to change, and varying acceptance by faculty and students [15]. The TAM has emerged as a valuable theoretical framework for examining these adoption challenges [10,16,17], yet its application within medical education contexts remains fragmented and inconsistently synthesized [16,18]. This knowledge gap may hinder evidence-based decision-making on the use of education technology that could enhance teaching and learning outcomes in medical education.

To address this limitation, this systematic review aims to synthesize the current research on the application of the TAM in medical education to provide insights into the factors influencing technology acceptance among medical professionals and students. The guiding questions that we aim to answer with this systematic review are as follows:

1. What is the state of TAM in medical education?
2. How has TAM been operationalized?
3. What education interventions are used in such studies?

## Methods

This review was designed and is reported using the PRISMA (Preferred Reporting Items for Systematic Reviews and Meta-Analyses) guidelines [19].

### Search Strategy

A systematic search was conducted in February 2024 to identify original published articles on TAM and medical education from January 2003 to December 2023. We set the search criteria to focus on the last 20 years to capture only the most recent advancements in the field. An author (JYT) then systematically searched 5 databases accessible through the university library. A search of “all fields” with the keywords “TAM” or “Technology Acceptance Model” and “Medical Education” was used for the Embase, MEDLINE, PubMed, PsycINFO, and Web of Science databases.

### Inclusion and Exclusion Criteria

Peer-reviewed articles were included if they used the TAM as a survey instrument in the study methodology or as a theoretical framework in medical education. This includes using the original TAM model proposed by Davis [20] or the TAM2 model proposed by Venkatesh and Davis [13]. We define medical education–related studies as training medical professionals, residents, and students pursuing their undergraduate, clerkship, postgraduate, or continuing medical education. If the study comprised a mix of medical students and students from other health care science professions (eg, nursing, pharmacy, emergency response), they were also included as part of the review.

Studies were excluded from our research if they were not related to medical education, such as research focused solely on nursing and allied health professions like pharmacy and physiotherapy, articles not written in English, articles published before 2003, and non–peer-reviewed documents, including theses or conference abstracts lacking comprehensive methodological details. When the cohort under study comprised a mixture of health professionals, including those who met the inclusion criteria, the entire cohort was included in the research analysis.

### Final Study Selection

After retrieving the search results from the identified database, JYT removed the duplicates and uploaded the articles into a shared Microsoft Teams [21] folder. The shortlisted articles were entered into an Excel spreadsheet for screening by the authors. The final screening process involved JYT noting articles for inclusion or exclusion based on the title or abstract, which was verified independently by the primary author (JWYL). JYT also assigned a reason for exclusion for each excluded article. In cases of uncertainty, the articles in question were retained and screened together by both authors (JWYL and JYT). JYT extracted the full text of the retained articles and this was verified by JWYL for consistency.

### Data Extraction and Analysis

After the shortlisted studies were identified, details of the studies were entered into the spreadsheet, including (1) general study information (eg, authors, title, and publication year), (2) participant-related information, (3) sample size, (4) application of the TAM framework, (5) study design, (6) statistical analysis used, (7) education intervention investigated, and (8) study quality (Medical Education Research Study Quality Instrument [MERSQI] score). Please see Table 1 for information gathered from the shortlisted studies.

**Table 1.** Articles included in the study.

Authors	Paper title	Publication year	Country	Study participants	Sample size	TAM^a^ application	Study design	Statistical analysis	Education intervention	MERSQI^b^ score
Wong G et al (2010) [22]	Internet-based medical education: a realist review of what works, for whom and in what circumstances	2010	United Kingdom	N/A^c^ (systematic review)	N/A	Research framework	Qualitative	N/A	E-learning	N/A
McGowan BS et al (2012) [23]	Understanding the factors that influence the adoption and meaningful use of social media by physicians to share medical information	2012	United States	Health care professionals	485	Survey instrument	Quantitative	Correlation	E-learning	9.5
Knight JF (2013) [24]	Acceptability of video games technology for medical emergency training	2013	Denmark	Health care professionals	37	Survey instrument	Quantitative	Multiple regression	Serious game	12
Fang TY et al (2014) [25]	Evaluation of a haptics-based virtual reality temporal bone simulator for anatomy and surgery training	2014	Taiwan	Medical undergraduates and health care professionals	14	Survey instrument	Quantitative	*t* test	Haptic device	9
Briz-Ponce L and Garcia-Penalvo F (2015) [12]	An empirical assessment of a Technology Acceptance Model for apps in medical education	2015	Spain	Medical undergraduates and health care professionals	124	Survey instrument	Quantitative	SEM (CB)^d^	Mobile learning	10
Ryan JR et al (2015) [26]	Ventriculostomy simulation using patient-specific ventricular anatomy, 3D printing, and hydrogel casting	2015	United States	Medical undergraduates	10	Survey instrument	Quantitative	Descriptive	3D printing	7
Huang HM et al (2016) [27]	Exploring learner acceptance of the use of virtual reality in medical education: a case study of desktop and projection-based display systems	2016	Taiwan	Medical undergraduates	230	Survey instrument	Quantitative	Correlation	Virtual reality	10.5
Briz-Ponce L et al (2017) [28]	Learning with mobile technologies — students’ behavior	2017	Spain	Medical undergraduates	124	Survey instrument	Quantitative	SEM (PLS)^e^	E-learning	9
Tahamtan I et al (2017) [29]	Factors affecting smartphone adoption for accessing information in medical settings	2017	Iran	Medical undergraduates	112	Survey instrument	Mixed	SEM (CB)	Mobile learning	10
Yeom S et al (2017) [30]	Factors influencing undergraduate students’ acceptance of a haptic interface for learning gross anatomy	2017	Australia	General undergraduates	89	Research framework	Quantitative	Descriptive	Haptic device	10
Basoglu N et al (2018) [31]	Exploring adoption of augmented reality smart glasses: applications in the medical industry	2018	Turkey	Medical undergraduates and health care professionals	71	Survey instrument	Quantitative	SEM (PLS)	Augmented reality	9
Duch Christensen M et al (2018) [32]	Learners’ perceptions during simulation-based training: an interview study comparing remote versus locally facilitated simulation-based training	2018	Denmark	Health care professionals	21	Research framework	Qualitative	N/A	Simulation-based training	N/A
Barteit S et al (2019) [17]	Technology acceptance and information system success of a mobile electronic platform for nonphysician clinical students in Zambia: prospective, nonrandomized intervention study	2019	Zambia	Medical undergraduates and health care professionals	109	Survey instrument	Quantitative	Correlation	E-learning	9
Chan KS and Zary N (2019)[33]	Applications and challenges of implementing artificial intelligence in medical education: integrative review	2019	United Arab Emirates	N/A (systematic review)	N/A	Research framework	Qualitative	N/A	Artificial intelligence in medical education	N/A
Johnson EM and Howard C (2019) [34]	A library mobile device deployment to enhance the medical student experience in a rural longitudinal integrated clerkship	2019	United States	Medical undergraduates	9	Survey instrument	Mixed	Descriptive	Mobile learning	9
Abdekhoda M et al (2020) [35]	A conceptual model of flipped classroom adoption in medical higher education	2020	Iran	Medical undergraduates	110	Survey instrument	Quantitative	Correlation	Teaching approach	11
Kucuk S et al (2020) [36]	A model for medical students’ behavioral intention to use mobile learning	2020	Turkey	Medical undergraduates	376	Survey instrument	Quantitative	SEM (CB)	Mobile learning	10
Lee CW et al (2020) [37]	User experience evaluation of the EPAs-based e-portfolio system and an analysis of its impact	2020	Taiwan	Health care professionals	20	Research framework	Qualitative	N/A	E-learning	N/A
Jeyakumar T et al (2021) [38]	Best practices for the implementation and sustainment of virtual health information system training: qualitative study	2021	Canada	Health care educators	18	Research framework	Qualitative	N/A	E-learning	N/A
Lee SS et al (2021) [39]	Mobile learning in clinical settings: unveiling the paradox	2021	Singapore	Health care professionals	171	Research framework	Mixed	Descriptive	Mobile learning	8.5
Zalat MM et al (2021) [40]	The experiences, challenges, and acceptance of e-learning as a tool for teaching during the COVID-19 pandemic among university medical staff	2021	Egypt	Health care professionals	346	Survey instrument	Quantitative	Descriptive	E-learning	8.5
Almarzouqi A et al (2022) [41]	Prediction of user’s intention to use metaverse system in medical education: a hybrid SEM-ML learning approach	2022	United Arab Emirates	General undergraduate and postgraduate	1858	Survey instrument	Quantitative	SEM (PLS)	E-learning	12
Bhardwaj M et al (2022) [42]	Perceptions and experience of medical students regarding e-learning during COVID-19 lockdown- a cross-sectional study	2022	India	Medical undergraduates	340	Research framework	Quantitative	Descriptive	E-learning	9
Bianchi I et al (2022)[43]	AnemiaAR: a serious game to support teaching of haematology	2022	Brazil	Medical undergraduates	14	Survey instrument	Quantitative	*U* test	Serious game	8
Chan E et al (2022) [44]	Medical teachers’ experience of emergency remote teaching during the COVID-19 pandemic: a cross-institutional study.	2022	Hong Kong	Health care educators	139	Research framework	Quantitative	Correlation	E-learning	9.75
Do DH et al (2022) [10]	Drivers of iPad use by undergraduate medical students: the Technology Acceptance Model perspective	2022	Canada	Medical undergraduates	834	Survey instrument	Quantitative	SEM (PLS)	Mobile learning	10.5
Harmon DJ et al (2022) [45]	Development and assessment of an integrated anatomy mobile app	2022	United States	Medical undergraduates	195	Survey instrument	Quantitative	SEM (CB)	Mobile learning	10
Komuhangi A et al (2022) [46]	Predictors for adoption of e-learning among health professional students during the COVID-19 lockdown in a private university in Uganda	2022	Uganda	Health science undergraduates	109	Survey instrument	Quantitative	Regression	E-learning	10
Lau V and Greer M (2022) [47]	Using technology adoption theories to maximize the uptake of e-learning in medical education	2022	United States	N/A (systematic review)	N/A	Research framework	Qualitative	N/A	E-learning	N/A
Bugli D et al (2023) [48]	Training the public health emergency response workforce: a mixed-methods approach to evaluating the virtual reality modality	2023	United States	Health care professionals	100	Survey instrument	Quantitative	Correlation	Virtual reality	9
Young Y et al (2023) [49]	Improving transitions between clinical placements	2023	United Kingdom	Medical undergraduates	19	Research framework	Qualitative	N/A	Website	N/A
Sallam M et al (2023) [50]	Assessing health students’ attitudes and usage of ChatGPT in Jordan: validation study	2023	Jordan	General undergraduates	458	Survey instrument	Quantitative	Correlation	Artificial intelligence in medical education	11
Cabero-Almenara J et al (2023) [51]	Degree of acceptance of virtual reality by health sciences students	2023	Spain	Health science undergraduates	136	Survey instrument	Quantitative	Regression	Virtual reality	10
Ndlovu K et al (2023) [52]	Evaluating the feasibility and acceptance of a mobile clinical decision support system in a resource-limited country: exploratory study	2023	Botswana	Health care professionals	28	Survey instrument	Mixed	Descriptive	Mobile learning	7
Lin CW et al (2023) [53]	Crowdsource authoring as a tool for enhancing the quality of competency assessments in healthcare professions	2023	Taiwan	Health care educators	50	Survey instrument	Quantitative	Correlation	E-learning	11
Rahadiani P et al (2023) [54]	Use of H5P interactive learning content in a self-paced MOOC [massive open online course] for learning activity preferences and acceptance in an Indonesian medical elective module	2023	Indonesia	Health science undergraduates	126	Survey Instrument	Quantitative	Correlation	E-learning	11
De Ruyck O et al (2024) [55]	A comparison of three feedback formats in an ePortfolio to support workplace learning in healthcare education: a mixed method study	2023	Belgium	Health care professionals	85	Survey instrument	Mixed	Correlation	E-learning	7

a TAM: Technology Acceptance Model.

b MERSQI: Medical Education Research Study Quality Instrument.

c N/A: not applicable.

d SEM (CB): covariance-based structural equation modeling.

e SEM (PLS): partial least squares structural equation modeling.

To assess the study quality of quantitative studies, the MERSQI was used to measure the methodological quality of the selected studies [56]. The MERSQI is an instrument that measures the quality of experimental, quasi-experimental, and observational studies. The MERSQI contains 6 domains (study design, sampling, type of data, validity evidence for the evaluation instrument, data analysis, and outcomes), with a study scoring a possible total of 18. The MERSQI was not designed for use in qualitative studies. Therefore, these studies will not be assessed using the MERSQI.

## Results

### Overview

**Figure 1.**
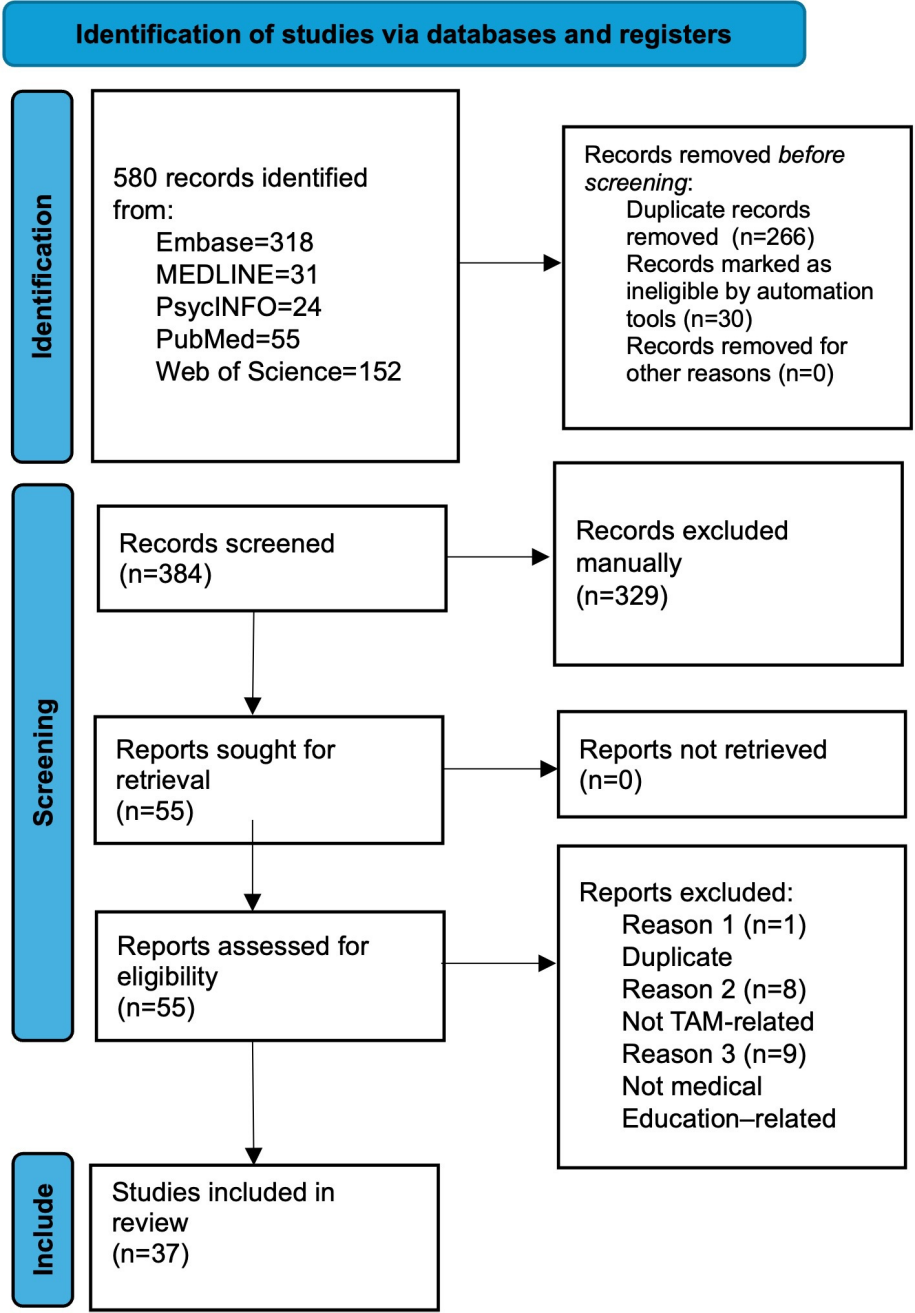
PRISMA flowchart. PRISMA: Preferred Reporting Items for Systematic Reviews and Meta-Analyses.

A systematic literature retrieval and analysis was methodically executed across 5 authoritative databases (Embase, MEDLINE, PsycINFO, PubMed, and Web of Science), yielding 580 records. The PRISMA checklist informed the review protocol and is depicted in the flow diagram in Figure 1. Using automation tools to narrow the search criteria, 30 studies were removed, and 266 duplicate records were excluded. This resulted in 384 studies that were eligible for screening. Based on the abstract or title, 329 studies that did not meet the study inclusion criteria were eliminated. This left 55 studies for full-text retrieval. Both authors read through all shortlisted papers and excluded a further 18 papers, where 1 was a duplicate, 8 did not use the TAM in the study, and 9 were unrelated to medical education. Therefore, the total number of studies included was 37.

Table 2 presents the source database, publisher, and number of studies found in the search results.

**Table 2.** Databases and search results (N=680).

Database	Vendor/publisher	Search results, n (%)
Embase	Elsevier	318 (46.3)
MEDLINE	OvidSP	31 (4.6)
PsycINFO	APA	24 (3.5)
PubMed	PubMed	155 (22.8)
Web of Science	Clarivate	152 (22.4)

### Year of Publication

**Figure 2.**
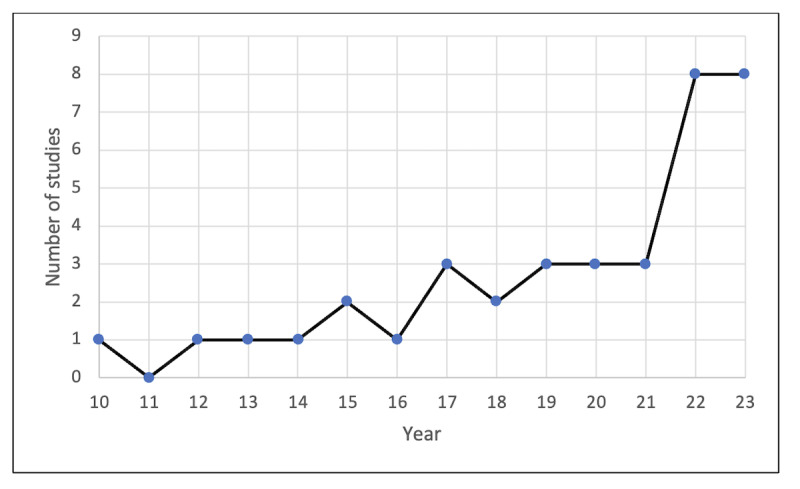
Number of publications by year from 2010 to 2023.

Despite the TAM being developed in the 1990s and our search spanning from 2003 to 2023, we only found a single study from 2010, which marked the earliest TAM usage in this review. The adoption of TAM in medical education remained relatively rare up to 2016. It was not until 2017 that a consistent uptick in the number of peer-reviewed publications using this model could be observed, with an average of 3 studies each year until 2022, when there was an almost 3-fold increase to 8 studies, which remained constant in 2023 (Figure 2). This surge in numbers was likely driven by the rapid integration of technologies from 2021 onward, which will be covered in the Discussion section.

### Country of Study

The included studies were conducted in 21 countries, with no single region dominating the publications. Six of the studies were conducted in the United States [23,26,34,45,47,48], 4 in Taiwan [25,27,53], 3 in Spain [12,28,51]; 2 each in Canada [10,38], Denmark [24,32], Iran [29,35], Turkey [31,36], United Arab Emirates [33,41], and the United Kingdom [22,49]; and 1 each in Australia [30], Belgium [55], Botswana [52], Brazil [43], Egypt [40], Hong Kong [44], India [42], Indonesia [54], Jordan [50], Singapore [39], Uganda [46], and Zambia [17]. This diverse set of countries suggests that TAM has been applied globally across low-income and high-income nations, reflecting its adaptability to various education and technological contexts. Table 3 is a description of the countries by location.

**Table 3.** Location of study by country.

Country	Number of studies
Australia	1
Belgium	1
Botswana	1
Brazil	1
Canada	2
Denmark	2
Egypt	1
Hong Kong	1
India	1
Indonesia	1
Iran	2
Jordan	1
Singapore	1
Spain	3
Taiwan	4
Turkey	2
Uganda	1
United Arab Emirates	2
United Kingdom	2
United States	6
Zambia	1

### Study Participants

The array of participants in the studies analyzed is quite diverse, reflecting the multifaceted nature of medical education. The review included 2 studies on general undergraduates of various disciplines [50], 1 on general undergraduate and postgraduate students of various disciplines [41], 3 on health care educators [38,44,53], and 9 on health care professionals [23,24,32,39,40,48,52,55,37]. Three studies centered around health science undergraduates [46,51,54], 12 studies focused on undergraduate medical students [10,26-29,34-36,42,43,45,49], and 4 investigated undergraduate medical students and health care professionals [12,17,25,31]. Notably, 3 studies were systematic or scoping reviews [22,33,47], which, by their nature, did not involve direct study participants. Table 4 presents a summary of publications by study participants.

**Table 4.** Summary of publications by study participants.

Study participants	Publication count
General undergraduates	2
General undergraduate and postgraduate students	1
Health care educators	3
Health care professionals (doctors, nurses, pharmacists, residents)	9
Health science undergraduate students	3
Medical undergraduates	12
Medical undergraduates and health care professionals	4
Review articles (scoping or systematic review)	3

### Application of TAM

The TAM served dual purposes in the surveyed studies. In 26 (70%) of the studies, it functioned as a survey instrument, quantitatively measuring the variables influencing user acceptance of and interaction with educational technology. The remaining 11 (30%) studies incorporated TAM as a foundational research framework, which involved thematic analysis of the collected data or shaping the methodology for data collection. This 2-pronged application of the TAM highlights its adaptability and role in the empirical and theoretical examination of technology adoption in medical education. Table 5 is a summary of the application of TAM.

**Table 5.** Summary of the applications of the Technology Acceptance Model.

Application	Count
Research framework	11
Survey instrument	26

### Study Design

The studies reviewed encompass quantitative, qualitative, and mixed methods research methodologies, each engaging the TAM differently. The quantitative studies operationalize the TAM through survey instruments, measuring variables such as perceived ease of use and perceived usefulness to explain the users’ behavioral intentions and actual technology use. In contrast, qualitative studies contextualize the TAM within the broader theoretical landscape, using it to guide the thematic analysis of focus group discourse or to underpin systematic reviews that explore the factors influencing technology adoption. The mixed methods approach combines both, where survey data are analyzed quantitatively while concurrently using qualitative techniques such as semistructured interviews or textual analysis to capture the subtleties of user experience and perception. Most of the included studies (25/37) were quantitative, 7 were qualitative, and 5 adopted a mixed methods approach, as described in Table 6.

**Table 6.** Summary of study methodology.

Methodology	Count
Quantitative	25
Qualitative	7
Mixed method	5

### Statistical Analysis

Correlation analysis was the predominant quantitative technique used in 10 studies to delineate the degree and direction of the linear relationship between the variables of interest. The next most used approach was structural equation modeling (SEM), with an equal number of studies (n=4) that used the covariance-based structural equation model and partial least squares structural equation model. Descriptive analysis was the third most frequently used method, implemented in 7 studies to succinctly summarize and describe the collected survey data. Three studies used regression analysis to predict the effect of the dependent variable based on the independent variable, and 2 other studies leveraged hypothesis testing, specifically the *t* test and *U* test, to conduct a comparative analysis of survey outcomes across different intervention groups. Seven studies were qualitative and therefore did not include statistical analysis. Table 7 summarizes the statistical approach taken by the reviewed studies, sorted by the complexity of the analysis.

**Table 7.** Statistical analysis approach of reviewed studies.

Statistical analysis approach	Count
Correlation analysis	10
SEM-CB	4
SEM-PLS	4
Descriptive analysis	7
Regression analysis	3
Hypothesis testing	2
Not applicable	7

### Types of Education Intervention

The studies reviewed can be classified broadly into 2 categories of education interventions: education technologies and education methodologies. Under education technologies, 1 study examined 3D printing [26], 2 studies examined artificial intelligence [33,50], 3 studies focused on virtual reality [27,48,51], and 1 on augmented reality smart glasses [31], indicating an interest in integrating cutting-edge approaches into medical education. E-learning emerged as the most prevalent intervention, with 15 studies emphasizing digital learning [12,17,22,23,37,38,40-42,44,46,47,53-55]. In comparison, using mobile devices for learning was explored in 8 studies [10,28,29,34,36,39,45,52]. Two studies each investigated the use of serious games [24,43] and haptic devices [25,30]. One study evaluated the use of a website to improve transitions between clinical placements [49]. Lastly, under the category of education methodologies, 1 study explored remote simulation training [34] and another explored flipped learning [45] in medical education. Table 8 summarizes the types of interventions investigated in the reviewed studies.

**Table 8.** Breakdown of the types of interventions investigated.

Intervention	Count
**Education technologies**	
3D printing	1
Artificial intelligence	2
Augmented/virtual reality	4
E-learning	15
Haptic device	2
Mobile device	8
Serious games	2
Website	1
**Education methodologies**	
Remote simulation training	1
Flipped learning	1

### Study Quality

**Figure 3.**
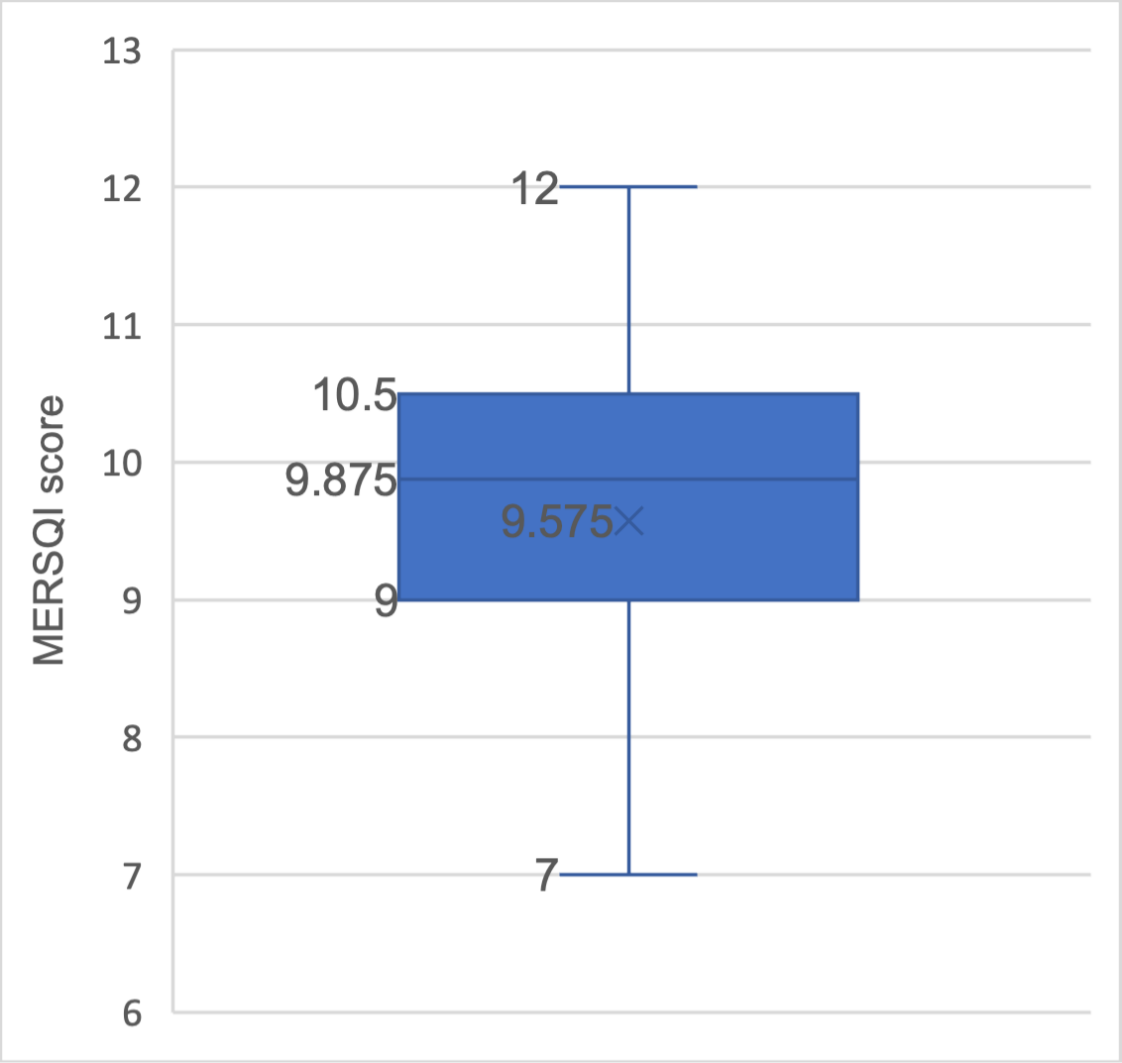
Boxplot diagram of the MERSQI score of reviewed studies. MERSQI: Medical Education Research Study Quality Instrument.

The MERSQI can be used to evaluate study quality in medical education research as it provides a validated, comprehensive framework for assessing methodological rigor across multiple dimensions. The MERSQI is a validated tool [57] consisting of 10 items across 6 domains: study design, sampling, data type, instrument validity, data analysis, and outcomes. Each domain can be scored up to 3, bringing the maximum score to 18. Thirty studies (25 quantitative and 5 mixed methods studies) were scored by the researchers using the MERSQI. The minimum score for the reviewed papers was 7, while the maximum was 12. The mean score was 9.58 (SD 1.31), with the mixed methods studies scoring generally below the mean score. Figure 3 is a boxplot diagram of the reviewed studies. Qualitative studies were not measured using the MERSQI. Table 1 displays a detailed summary of the MERSQI scores of the studies reviewed.

## Discussion

### Opportunities for TAM in Medical Education

Over the past two decades, technological progress has significantly shaped the education landscape. Traditional teaching approaches are enhanced with technology, making learning no longer bound by space or time. Yet, this systematic review found that the number of studies in medical education that use the TAM is notably infrequent when contrasted with other fields such as health informatics (134 studies) [16], higher education (104 studies) [58], mobile learning (87 studies) [59], and health profession education (142 studies) [18]. The review found a modest output of 1 study per year from 2010 to 2016. There was an uptick of 3 publications per year through 2021, followed by a large increase to 8 studies in 2022 and 2023, suggesting a growing interest in and recognition of TAM’s relevance in medical education research.

This could be because the health care education field takes a conservative approach when adopting new digital initiatives. The curricula in medical education are highly structured and content-heavy, thus leaving little room for incorporating digital technologies. However, more recently, there have been calls for reforms within the curriculum [60], especially to integrate technology to enhance students’ learning experience [61,62], which has been shown to affect student learning outcomes positively [63]. This can explain the steady increase in the number of studies that use TAM to understand user acceptance of learning interventions.

The low adoption rate of the TAM within medical education may be due to the focus on prioritizing satisfaction and basic usage statistics [64-66] when evaluating new technologies for learning. This approach overlooks the more nuanced dimensions that TAM examines, such as perceived usefulness and perceived ease of use. This emphasis on program-level satisfaction metrics fails to capture the complex psychological and organizational factors influencing technology acceptance in health care educational environments. Such reliance on superficial evaluation matrices creates a significant gap between measuring program satisfaction and truly understanding the complex factors driving technology acceptance and sustained use in medical education.

The COVID-19 pandemic caused a global shift to digital platforms for learning. This created an urgent need to understand technology adoption in education and health care. The TAM became a framework for evaluating user acceptance of rapidly implemented technologies like e-learning platforms [26,34,56] and mobile learning [19-21]. The forced accelerated adoption of mobile and web-based learning highlighted the importance of TAM in assessing factors such as perceived usefulness and ease of use for remote teaching tools. The pandemic served as a global natural experiment in technology adoption, driving researchers to apply TAM across diverse contexts to address barriers to digital transitions. This surge in TAM applications demonstrated its adaptability in analyzing critical acceptance factors [32,36,42,46] during systemic disruptions, offering insights into user behavior that were essential for navigating the rapid technological transformations brought on by the crisis.

In this systematic review, each study with a quantitative element was appraised using the MERSQI, which is designed to assess the quality of published medical education research. Typically, a higher score is often associated with greater methodological rigor and would result in higher acceptance to quality journals [67,68]. With a mean MERSQI score of 9.6 (SD 1.17), the average score found within this review was higher than that found in a paper by Smith and Learman [57], yet it did not reach the benchmark of the high-quality score of 10.5 (SD 2.5) described by Reed et al [67]. Our analysis indicates that the substantial scores in this review are partly due to the inclusion of the TAM. Given that the TAM is a validated survey tool, its use—whether in its original or modified version—immediately contributes to a base score of five: 3 points for the tool’s validity and 2 points for measuring behavioral outcomes. A study can accrue 4-5 points by using a methodologically robust and sound approach in the study design and reporting. Therefore, incorporating the TAM may potentially contribute to a higher quality of publication output.

### Operationalizing the TAM

The TAM was originally developed as a theoretical framework based on the Theory of Planned Behavior [69], which can be operationalized as a survey based on the constructs within the model. This review found that the prevalent application of TAM in studies is through survey instruments, aligning with findings from other reviews [59]. Apart from the survey instrument, the TAM can be used qualitatively, such as adapting the constructs to guide the discourse in focus group discussions [32] or semistructured interviews [39].

Several different statistical analysis approaches were used to analyze the qualitative data. SEM stands out as one of the most comprehensive methods, adept at testing hypotheses concerning both observed and latent variables [70]; these studies generally had higher MERSQI scores [10,12,29,36,41,45]. Despite its robustness, SEM demands a thorough grasp of complex statistical concepts and a sufficiently large sample size to ensure the stability and accuracy of its estimates [71]. Correlation analysis offers a more straightforward approach to measuring the strength and direction of relationships between variables. Regression analysis further extends the analytical capability by providing predictive insights and facilitating the exploration of potential causal links between factors. Additionally, the TAM is frequently used in a descriptive capacity, offering an interpretive lens to dissect and articulate the intricacies of user interactions with technology, their attitudes, and the behavioral intentions that these factors precipitate.

This systematic review found that a large number of learning interventions were investigated for e-learning. This could be explained by the shift in higher education over the past 2 decades to web-based learning [72], accelerated by the COVID-19 pandemic, which necessitated and expedited the transition to web-based learning across various disciplines [73], including medical education. The integration of mobile technology into our everyday lives has naturally extended into the realm of education. This has prompted research on mobile devices for information access [15,23,32,47] and mobile apps for learning [12,45].

Additionally, this systematic review found studies delving into more innovative educational technologies beyond e-learning, such as virtual reality, augmented reality, serious games, and 3D printing. Virtual reality allows for students to practice their technical skills repeatedly in a risk-free setting, thus increasing their confidence and proficiency without jeopardizing patient safety [25,74]. Augmented reality overlays digital information onto physical or live environments, allowing students to understand complex anatomical structures [6] and to gain spatial awareness [75]. Another emerging tool that combines interactive gameplay with educational outcomes is serious games, which simulate real-world medical scenarios in a controlled and engaging environment [24,43]. 3D printing allows for the rapid development of customized models that can be used for teaching and learning [26]. Together, these technologies are changing the way learning is happening in medical education by providing immersive, interactive, and accessible tools to complement traditional teaching approaches. The TAM can serve as a valuable framework for understanding how these new and existing technologies are adopted and used for learning in medical education. By having a framework, educators and institutions can use the TAM to evaluate the integration of these technologies into their curricula and their potential for improving educational outcomes.

### Limitations and Future Research

One limitation of this review is that it only encompasses data published up until 2023. Given the observed publication trend, it is plausible that subsequent studies using TAM in 2024 and beyond fall outside the scope of this review. Consequently, the conclusions drawn here are pertinent to the specified research period. Future studies should consider extending the review to include these additional years, thereby capturing a more comprehensive dataset, potentially offering a more current evaluation of TAM’s application in the field.

Although this review contributes valuable insights into the use and application of TAM in education, the findings are primarily within the context of medical education and exclude other health professions, including nursing and allied health professionals. Medical education is a highly specialized domain with unique challenges and practices that may not directly translate to the broader educational context outside of medicine. Furthermore, the complexity and heterogeneity within medical education, such as the variation in curricula and culture, may pose an additional challenge to generalizing findings even within the discipline.

Despite these limitations, the framework used in this review offers significant potential for broader application across other health care disciplines. Researchers could adapt this approach to explore TAM’s adoption and effectiveness in nursing education, allied health training, or interdisciplinary health care programs. Expanding research beyond medical education would enhance the generalizability of findings and provide comparative insights into how TAM influences technology adoption across diverse health care professions. Additionally, extending TAM-based research to non–health care fields could further enrich our understanding of its applicability and utility in varied educational contexts.

Future researchers should consider adopting the additional constructs in TAM2 to better understand how social and cognitive factors influence technology acceptance beyond the perceived ease of use and perceived usefulness. For example, researchers can investigate how subjective norms or job relevance may influence the students’ willingness to adopt new technologies and professional identities in an educational context.

### Conclusions

This systematic review aimed to understand the use of the TAM in medical education over the past two decades, highlighting its utility as both a theoretical framework and survey instrument. This study reported on TAM’s increasing popularity and versatility for measuring and understanding the learners’ acceptance of the intervention. With the increasing integration of e-learning, digital learning, and other new learning modalities, it is critical that researchers can leverage technologies that learners will adopt. Such curriculum innovations are critical for maintaining educational continuity in the face of global health challenges by facilitating remote learning and continuous professional development. Consequently, these curricular reforms are expected to catalyze a significant surge in the adoption of digital technologies within medical education.

The growing importance of the TAM in understanding technology acceptance cannot be overstated, especially in medical education, where the use of artificial intelligence, virtual reality, and other adaptive learning platforms is increasingly popular. Educators and developers can use TAM as a theoretical framework to design curricula or interventions considering barriers to adoption, such as organizational support or the intervention’s technical complexity. The TAM is relevant as an evaluation tool and can guide future innovations in medical education. Policymakers should consider using the insights gained using the TAM to develop strategies for the education system while meeting the challenges of cost, accessibility, and infrastructure development.

This review provides a comprehensive understanding of how the TAM has been applied within the field of medical education over the past 20 years. As the field continues to innovate, TAM will continue to play an important role in helping educators, policymakers, and researchers understand the dynamics of technology integration and the impact on teaching and student learning outcomes.

## Supplementary material

10.2196/67873 Checklist 1 PRISMA checklist. PRISMA: Preferred Reporting Items for Systematic Reviews and Meta-Analyses.

## Abbreviations

MERSQI: Medical Education Research Study Quality Instrument
PRISMA: Preferred Reporting Items for Systematic Reviews and Meta-Analyses
SEM: structural equation modeling
TAM: Technology Acceptance Model
