# Supplementary figures and images for: Technology Acceptance Model in Medical Education: Systematic Review
Source: JMIR Med Educ. 2025 Jul 16;11:e67873. doi: 10.2196/67873 (PMC12285687; doi:10.2196/67873)

Figure 1. PRISMA flowchart.

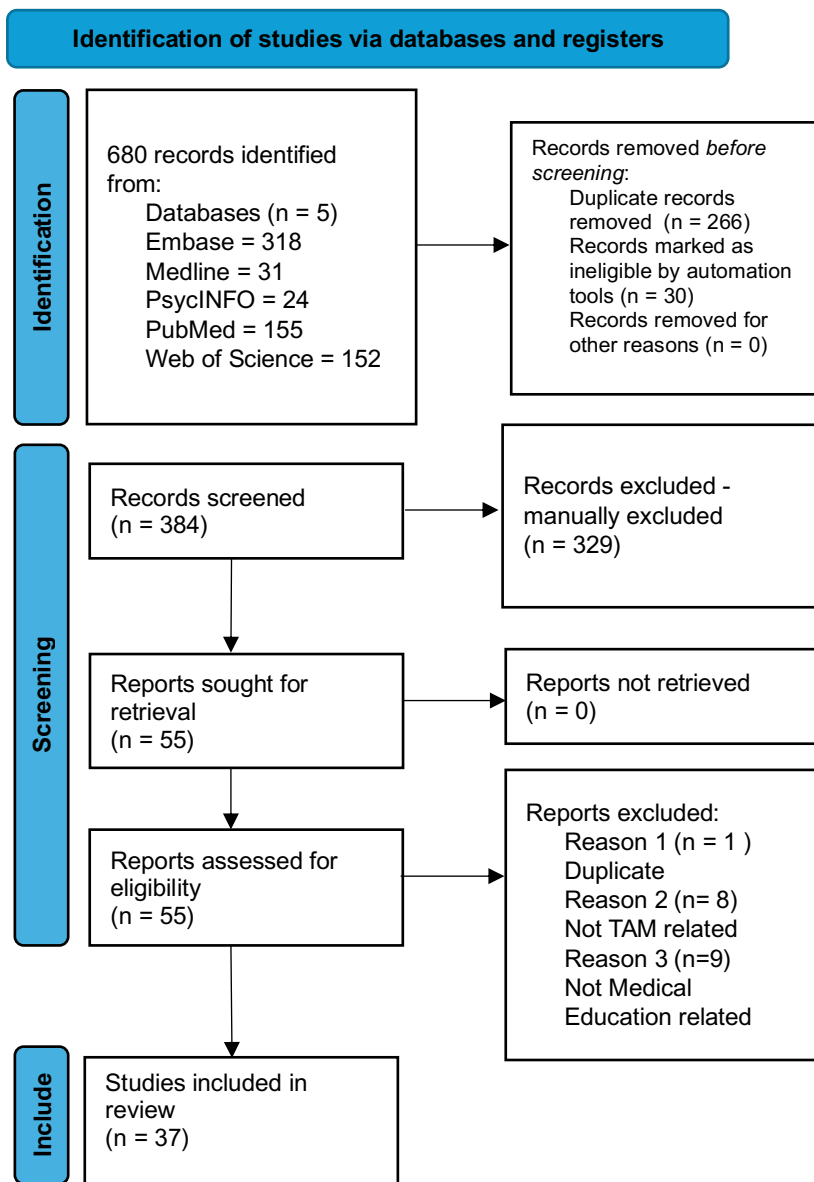

Supplement: Checklist 1 [file mededu-v11-e67873-s001.pdf]
